# 929. The different characteristics in renal impairment between severe fever with thrombocytopenia syndrome and hemorrhagic fever with renal syndrome in South Korea

**DOI:** 10.1093/ofid/ofad500.974

**Published:** 2023-11-27

**Authors:** Sang Hyun Ra, Hyeonwoo Kwon, Geonui Kim, Seongman Bae, Eui Jin Chang, Jiwon Jung, Min Jae Kim, Yong Pil Chong, Sang-Oh Lee, Sang-Ho Choi, Yang Soo Kim, Sung-Han Kim

**Affiliations:** Asan Medical Center, Songpa-gu, Seoul-t'ukpyolsi, Republic of Korea; Asan Medical Center, Songpa-gu, Seoul-t'ukpyolsi, Republic of Korea; Asan Medical Center, Songpa-gu, Seoul-t'ukpyolsi, Republic of Korea; Asan Meidical Center, Songpa-gu, Seoul-t'ukpyolsi, Republic of Korea; Department of Internal Medicine, Asan Medical Center, Seoul, Korea, Seoul, Seoul-t'ukpyolsi, Republic of Korea; Asan Medical Center, Songpa-gu, Seoul-t'ukpyolsi, Republic of Korea; Asan Medical Center, Songpa-gu, Seoul-t'ukpyolsi, Republic of Korea; Asan Medical Center, Songpa-gu, Seoul-t'ukpyolsi, Republic of Korea; Asan Medical Center, Songpa-gu, Seoul-t'ukpyolsi, Republic of Korea; Asan Medical Center, Songpa-gu, Seoul-t'ukpyolsi, Republic of Korea; Asan Medical Center, Songpa-gu, Seoul-t'ukpyolsi, Republic of Korea; Asan medical center, Seoul, Seoul-t'ukpyolsi, Republic of Korea

## Abstract

**Background:**

Severe fever with thrombocytopenia syndrome (SFTS) is an emerging tick-borne disease with high fatality rate, caused by SFTS virus (officially renamed as Dabie bandavirus). Hemorrhagic fever with renal syndrome (HFRS) is caused by various serotypes under the genus *Orthohantavirus* and transmitted by aerosolized excreta from rodents. Both have similar clinical manifestations characterized by high-grade fever, thrombocytopenia, hemorrhagic tendencies, and often lead to multiorgan dysfunction including kidneys. However, there are limited data on difference in renal impairment between SFTS and HFRS. We thus investigated the differential characteristics in renal manifestations between SFTS and HFRS in South Korea.

**Methods:**

All adult patients aged ≥ 18 years who were admitted to Asan Medical Center, a 2700-bed tertiary hospital in Seoul, South Korea between August 2005 and September 2022 were retrospectively enrolled. SFTS and HFRS were diagnosed by PCR and serologic confirmation, respectively. Patients with a history of previous urologic or chronic kidney diseases were excluded.

**Results:**

A total of 45 SFTS and 33 HFRS patients were enrolled. Proteinuria and hematuria at admission were observed in 25 (56%) and 19 (42%) for SFTS, and 29 (88%) and 27 (82%) for HFRS, respectively. The 12 (27%) SFTS patients and 26 (78%) HFRS patients initially showed acute kidney injury (AKI). The degree of the hematuria at admission and the presence of AKI were significantly associated with the severity of clinical course in patients with SFTS, not in HFRS (Table 1).

**Figure:**
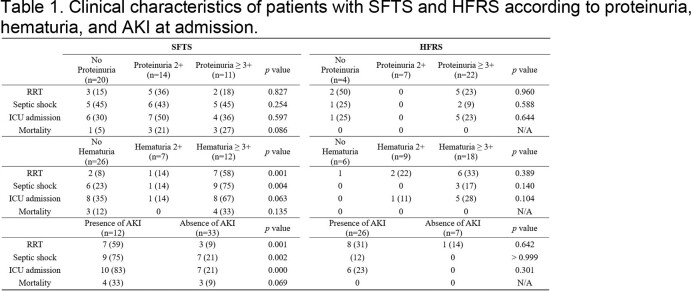

Abbreviation. AKI, acute kidney injury; HFRS, hemorrhagic fever with renal syndrome; ICU, intensive care unit; N/A, not assessed; RRT, renal replacement therapy; SFTS, severe fever with thrombocytopenia syndrome. Data represent number of patients (%) unless otherwise specified.

**Conclusion:**

Proteinuria and/or hematuria commonly occurred both in SFTS and HFRS. The presence of hematuria or AKI was associated with worse clinical course in SFTS, but not in HFRS. Thus, in the areas endemic to both diseases, repeated monitoring of kidney function test with urinalysis is required until these two diseases are differentiated.

**Disclosures:**

**All Authors**: No reported disclosures

